# Scarcity mindset’s positive association with using alternative financial services

**DOI:** 10.1371/journal.pone.0339127

**Published:** 2026-02-20

**Authors:** George Rooney, Cäzilia Loibl

**Affiliations:** Department of Human Sciences, The Ohio State University, Columbus, Ohio, United States of America; U of U: University of Utah, UNITED STATES OF AMERICA

## Abstract

The scarcity mindset, where people do not have what they feel that they need, can help consumers address the area of scarcity, but on the other hand can lead to neglect and mistakes elsewhere. This study tests whether a scarcity mindset is associated with the use of alternative financial services, whether the association is stable above and beyond established predictors of the use of alternative financial services, and whether it differs by household income. Data come from the 2021 FINRA National Financial Capability Study’s State-by-State survey wave, N = 24,349. Results of binary logistic regression analyses show that, controlling for established predictors of alternative financial services use and socio-demographic characteristics, a stronger scarcity mindset is significantly positively related to alternative financial services use. These findings hold for the pre-COVID-19 2018 data with regard to income groups, and although high-income respondent are lesser users of alternative financial services, the scarcity mindset transcends income status and emerges as a significant predictor of alternative financial services use for study participants regardless of household income. Study implications point to the need to assess the role of the scarcity mindset for newer alternative financial services products, including fintech applications. (192/300).

## Introduction

The scarcity mindset is commonly defined as the belief that one has less than one needs [1]. As it pertains to financial decisions, it reflects a person’s belief that he or she do not have the wherewithal to satisfy personal and household necessities and desires [2–4]. There is a small number of research studies related to the scarcity mindset and financial behaviors, typically situated among financially vulnerable population groups. Results have documented an association with short-term decision-making, focus on the source or manifestation of scarcity, attentional neglect of other needs and problems, and trade-offs [1, 5]. Less attention, however, has been paid to investigating whether a link exists between a scarcity mindset and decisions about which financial products and services to engage with. This perspective is important because the costs and risks associated with financial products and services, especially those outside the financial mainstream, have been linked to individual financial wellbeing [6]. These alternative financial services are the focus of the current research.

Alternative financial services represent financial products and services typically offered outside of the traditional banking sector. Alternative financial services include payday loans, car title loans, pawn shop loans/transactions, rent-to-own transactions, and nonbank check cashing service [7, 8]. These financial services are of interest to researchers, policymakers, and practitioners because they are less regulated, inefficient, costly, potentially riskier than products and services provided by the traditional banking sector, and targeted at low-income and financially-vulnerable consumers [8]. Their use in the United States is widespread and persistent. Latest data, from 2022, documented that 26.5% of households used overdrafting and insufficient funding services [9]. In addition, data from 2023, show that about 5.8% of households used rent-to-own services, payday loans, pawn shop loans, car title loans, and/or income tax refund advancement loans [10].

Users of alternative financial services typically have limited access to traditional banking products and services and are often unbanked [8]. The report found that use of these services by unbanked individuals is typically due to lack of or limitations around financial knowledge, financial education, access to financial counsel and advice, financial socialization, and financial inclusion [8]. Less information is available about psychological factors that may explain the use of alternative financial services. One study using data from the 2018 National Financial Capability Study (N = 20,644) found the use of alternative financial services was positive and significantly related to financial anxiety, and was positive and significantly related to financial anxiety when interacting with overconfidence in financial knowledge [11]. A small-sized survey of college students aged 18–30 years (N = 296) found that users of payday loans and/or car title loans showed more impulsivity and mental discounting compared to non-users (*p* = .023) [12].

The current research adds new insights about the role of psychological factors that may explain the use of alternative financial services. Our focus is the scarcity mindset, a powerful but lesser known factor in financial decision-making [1, 5]. We test whether the scarcity mindset is associated with the use of alternative financial services, using a national survey sample, as opposed to exclusively low-income individuals. Next, we examine whether the association between a scarcity mindset and alternative financial services usage is stable above and beyond established predictors of the use of alternative financial services. We control for individuals’ objective and subjective financial knowledge, willingness to take risk, and difficulty covering household expenses. Finally, we investigate whether the association of scarcity mindset and use of alternative financial services is stronger for individuals living in lower-income households, compared to mid- and higher-income households.

### Link between scarcity mindset and financial behaviors among financially vulnerable population groups

A number of studies have examined the scarcity mindset in the context of financially vulnerable populations, using both experimental and survey methods. The scarcity mindset, defined as an individual not having what they feel that they need [5], has been developed in the context of decision-making among low-income individuals [13, 14]. The theoretical framework is situated in the intersection of economics and psychology, in an effort to explain economic decisions and behavior among those in poverty that led to reinforce poverty [13]. The economic perspective suggests that the conditions of life in poverty prevents individuals from overcoming the barriers, referring to the wealth of research in the fields of labor economics [15], housing economics [16], and education economics [17]. The psychological perspective posits that certain personality traits are linked to poverty, relating it, for example, to research on the big-five personality traits as well as research on mental health [18, 19]. The framework of the scarcity mindset built on these perspectives by describing specifically how, “resource scarcity creates its own mindset, changing how people look at problems and make decisions.” (p. 682) [20]. The scarcity mindset framework is based on two proposition, that individuals will focus on problems where scarcity is most pronounced and, second, that this focus leads to lesser attention given to other problems [20]. As a result of both, the scarcity mindset can lead to decision mistakes, which can severely impact the financial security of individuals with limited means [13, 21, 22].

The most prominent experimental studies are by Shah, Mullainathan and Shafir [20] who conducted a series of small-scale experiments (4 experiments recruited participants using a crowdsourcing platform, and 1 experiment recruited university students) in which conditions of scarcity or abundance of resources, borrowing allowances, and cognitive load were induced. In each experiment, a form of the scarcity mindset was created; participants were randomly assigned to a poor or rich budget and randomly assigned with the ability to borrow or not. Also, in each of the experiments, attention and/or focus tasks were included, which added cognitive load in each fun game and allowed the researchers to observe the relationship between scarcity and attention/focus. The results show that a scarcity mindset is associated with greater focus and engagement with a problem at hand but can also be associated with neglectful behavior or mistakes, such as overborrowing. The study also found that a scarcity mindset created cognitive load by shifting attention to the scarcity at hand and less attention beyond this. [1] next reported on a series of experiments in the U.S., in which scarcity, as related to participants’ income or experimentally imposed scarcity, was associated with trade-off thinking in valuing items (e.g., electronic tablet) but less so with contextual factors (e.g., willingness of price to pay for beer at a resort or a grocery store). A replication of several of the Shah, Shafir and Mullainathan [1] studies with U.S. participants during the COVID-19 pandemic tested the role of threat perceptions on participants along with the original effects of contextual factors during financial scarcity [23]. The studies corroborated findings that contextual factors were weaker for individuals with a scarcity mindset during the time of COVID-19 [23]. These studies confirmed, however, a stronger tendency for financial and other decision-making mistakes related to trade-offs as a result of a scarcity mindset.

Focusing solely on the financial context, Cook and Sadeghein [24] conducted a series of four experiments using a crowdsourcing platform to examine the role of perceived scarcity on payday loan borrowing behavior, perceptions of risk, and ego effects. The study supplemented the analysis with qualitative data from the Consumer Financial Protection Bureau’s complaints database. When manipulating participants’ perceived scarcity situation using, for example, liquidity constraints, criticality of need, loss consequences, and borrowing options, the study found a negative relationship between perceived financial scarcity and sound financial analysis and decision-making [24].

Using a survey-based approach and the term “financial scarcity,” van Dijk, van der Werf and van Dillen [25] developed the “Psychological Inventory of Financial Scarcity” to measure a person’s subjective experience of financial scarcity. The Inventory includes four factors: a perceived threat of having a shortage of money, a lack of control of one’s financial situation, rumination and preoccupation with financial needs, and short- vs long-term focus and trade-offs. The Inventory was integrated in two waves of the Longitudinal Internet Studies for Social Sciences survey in the Netherlands in 2018 and 2020. Results showed evidence of a positive relationship between persons’ financial scarcity mindset and financial avoidance, which was defined as a person refraining from taking positive steps while experiencing financial stress and difficulties [2]. In online experiments with consumers in the United Kingdom, which manipulated the conditions to simulate a scarcity mindset in household-related tasks and finances, financial scarcity was correlated with an increase in discounting of losses and gains [26]. The Inventory overlaps in part with another scale, the Perceived Scarcity Scale [27], which also measures economic vulnerability [28]. The Perceived Scarcity Scale tests for a needs- and wants-based interaction among material, time, and psychological resource scarcity to explain how stress and health are related to socio-economic status [29].

Taken together, experimental research has identified the scarcity mindset to be associated with contextual focus and engagement with the source or manifestation of scarcity but also associated with mistakes and poor financial decision-making with non-contextual financial matters. Survey research has shown that a scarcity mindset is associated with financial avoidance (i.e., avoiding proactive financial decisions) and mental discounting of gains and losses. For the current study, we translate these findings into our working hypothesis, which proposes a direct association between a scarcity mindset and the use of risky financial products and services, such as alternative financial services use, above and beyond established predictors of alternative financial services use.

### Established predictors of alternative financial services use

The majority of the research on the use of alternative financial services has been conducted in the context of the financial capability framework, among those with financial challenges [8]. The financial capability framework is broad in scope and investigates the factors that help determine whether individuals can manage their finances and financial situation and participate in the financial system. These factors tend to focus on demographics, objective and subjective financial knowledge, willingness to take risk, ability to balance income and expenses [8]. A small number of studies extend beyond the financial capability framework to investigate psychological factors associated with alternative financial services use.

An early survey by Lawrence and Elliehausen [30] of payday loan customers, was among the first to profile the users of a particular alternative financial service. A telephone survey was conducted among 427 payday loan customers drawn from a sample of customers of companies in the Community Financial Services Association of America. Payday loan users tended to be low-to-moderate income, using payday loans mostly as a transitional product as they evolved through the economic life cycle. When faced with liquidity shortfalls and/or unexpected expenses and the reality or perception of few alternatives, consumers relied on payday loans to meet the shortfalls [30]. Over the years, the demographic characteristics have been mostly consistent. In a literature review study, higher likelihood of using alternative services has been associated with lower income, lower educational attainment, including financial education, male gender, ages in the 18–34 age bracket, non-White race, living with a partner marital status, and being a renter [31]. Some differences have been noted depending on the actual type of alternative financial service. For example, a survey found that payday loan borrowers were more often women and renters, whereas automobile title loan borrowers were more often men and evenly split among renters and homeowners [32, 33].

Going beyond the profiling of users, several studies have targeted the link between the use of alternative financial services and financial literacy. Lusardi, Schneider and Tufano [34] analyzed a U.S. subsample (N = 1,353) of the thirteen-country TNS Global Economic Crisis survey that was conducted to examine how consumers deal with household risk, defined as a modest financial shock (or a $2000 needed in next 30 days). Among coping strategies considered, several alternative financial services were evaluated, specifically, payday loans, payroll advance loans, and pawn shop transactions. The study documents an unexpectedly positive associated with financial literacy, specifically with financial education in school and having calculated the value of assets and debts. As expected, alternative financial services use was negatively associated with consumers having reviewed retirement statements and holdings and assessed how households’ financial holdings may change [34]. Lusardi and de Bassa Scheresberg [35] used the 2009 National Financial Capability Study to show that financial knowledge, measured with the “Big 3” questions, was significant and negatively related to use of alternative financial services, controlling for risk preferences, financial fragility, and financial inclusion (N = 22,464). In contrast, preferences for financial risks and financial fragility were positively associated with the use of alternative financial services [35]. Harvey [36] focused on young adults’ use of alternative financial services across three National Financial Capability Study waves (2009–2015) to show that taking a personal finance course in high school lowered their likelihood of using payday loans by 4 percentage points, contradicting earlier general-population findings.

Robb, Babiarz, Woodyard and Seay [37] were the first to shift focus and document that respondents high on the subjective financial knowledge scale (i.e., unjustifiably self-confident or overconfident) were most likely to use alternative financial services, while the reverse was, again, found for objective financial knowledge, by analyzing the combined 2009 and 2012 waves of the National Financial Capability Study (N = 53,655). Multiple analyses with different strata of respondents’ financial situation (e.g., owning a home; having health insurance) further documented bounded rationality as linked to alternative financial services use. Kim, Cho and Xiao [11] added that financial anxiety was related to greater use of alternative financial services and a higher number used, especially car title loans, payday loans, pawn shops, and rent-to-own arrangements were linked to greater financial anxiety, using the 2018 wave of the National Financial Capability Study (N = 20,644).

Taken together, evidence suggests an association of demographic groups that experience financial vulnerability, financial knowledge, risk tolerance, and financial stress with alternative financial services use. The rationale for linking the scarcity mindset to alternative financial services usage is that the feeling of not having enough can be associated with hyperfocus on this feeling and a search for relief. Alternative financial services, such as a payday loan or pawn shop transaction, can provide quick, relatively easy financial relief, but at a relatively high cost in the future. Our working hypothesis then is that a stronger and more pronounced scarcity mindset is related to a higher likelihood of poorer financial decisions, such as using high-cost alternative financial services. We expect the proposed relationship to hold above and beyond factors known currently to predict alternative financial services use, such as financial knowledge [38], willingness to take risk [39, 40], and vulnerability around finances and making ends meet [41, 42].

Based on this limited evidence, we expect a positive association of a scarcity mindset with alternative financial services use, while accounting for established cognitive and emotional factors and socio-demographic borrower characteristics. Our specific research questions are:

1. Is the scarcity mindset associated with the use of alternative financial services? We use binary logistic regression to predict usage with a newly developed scarcity mindset score for a 2021 sample of U.S. Americans.
2. Is the association between a scarcity mindset and alternative financial services usage stable above and beyond established predictors of the use of alternative financial services? We control for objective and subjective financial knowledge, willingness to take risk, and difficulty covering household expenses.
3. Is the association of scarcity mindset and use of alternative financial services stronger for adults living in lower-income households, compared to adults living in mid and higher-income households?

## Materials and methods

### Data

The study examines survey data of the 2021 National Financial Capability Study State-by-State Survey. This cross-sectional, online survey effort is funded by the Financial Industry Regulatory Authority, a government-authorized not-for-profit organization that regulates broker-dealers in the United States. [38]. The State-by-State Survey was first conducted in 2009, establishing a tri-annual survey schedule. The survey questionnaire remains in most parts the same across survey administrations. The State-by-State Survey collected responses from 27,118 U.S. American adults in 2021. Within each state, the sample size was set to approximate Census distributions for age by gender, ethnicity, education level, and income based on data from the Census Bureau’s American Community Survey [38]. The data underlying the results presented in the study are available from the FINRA Foundation [43]. The 2021 National Financial Capability Study (State-by-State Survey) was reviewed and determined as exempt from IRB review by Sterling IRB (IRB ID: 9067-GMottola, determination date: June 28, 2021; Category 2 Exemption (DHHS)).

We used listwise deletion to account for non-responses, prefer not to say, and don’t know. The final analytical sample in the data analysis consists of 24,349 (89.8%) responses in the 2021 survey. All statistical analysis was conducted with StataCorp Stata Version 18.0 [44].

### Variables

The variable “use of alternative financial services” is the focal outcome measure*.* Respondents were asked to indicate their usage of five different types of alternative financial services “In the past 5 years, how many times have you … 1) “Taken out an auto title loan? Auto title loans are where a car title is used to borrow money for a short period of time. They are NOT loans used to purchase an automobile; 2) Taken out a short term ‘payday’ loan?; 3) Gotten an advance on your tax refund? This is sometimes called a ‘refund anticipation check’ or ‘Rapid Refund’ (Not the same as e-filing); 4) Used a pawn shop?; and 5) Used a rent-to-own store?” The seven response options included: Never, 1 time, 2 times, 3 times, 4 or more times, Don’t know, and Prefer not to say. Following convention [11, 34], we created a dichotomous variable that is coded as 1 if respondents have used alternative financial services and 0 if they had not. An additional count variable ranges from 0 to 5 based on the number of alternative financial services that respondents have used.

### Predictor variables

The variable “scarcity mindset” is the main predictor variable*.* A proxy variable was constructed from the National Financial Capability Study State-by-State dataset, with an expert-based approach described in S1 File and in Rooney and Loibl [45]. Scarcity mindset is measured with the three measures, 1) Because of my money situation, I feel like I will never have the things I want in life; 2) I am just getting by financially; 3) I am concerned that the money I have or will save won’t last. The five response options included: does not describe me at all, describes me very little, describes me somewhat, describes me very well, describes me completely. Cronbach’s alpha was 0.87 in 2021. The responses were summed, resulting in a value between 3 and 15.

The variable “objective financial knowledge” was a count measure of six questions to generate a score. An example is, “A 15-year mortgage typically requires higher monthly payments than a 30-year mortgage, but the total interest paid over the life of the loan will be less. True or false.” Following convention [34, 35], correct responses were coded as 1 resulting in an objective financial knowledge score ranging from 0 (no correct answer) to 6 (all answers correct). The variable *“*subjective financial knowledge” was constructed based on responses to a single question, ”On a scale from 1 to 7, where 1 means very low and 7 means very high, how would you assess your overall financial knowledge?”

The variable *“*willingness to take financial risk” was measured with the single item, “When thinking of your financial investments, how willing are you to take risks? Please use a 10-point scale, where 1 means not at all willing and 10 means very willing.” The variable *“*difficulty covering monthly expenses” was measured with the item, “In a typical month, how difficult is it for you to cover your expenses and pay all your bills?”. Response options were (1) very difficult, (2) somewhat difficult, and (3) not at all difficult. A total of 43.6% reported this being very or somewhat difficult.

Demographic and socioeconomic control variables included gender (male, female (reference category)), race/ethnicity (White non-Hispanic (reference category), Black non-Hispanic, Hispanic, Asian/Pacific Islander non-Hispanic, Other non-Hispanic (American Indian, Other, multiple ethnicities)), age (continuous), marital status (married/living with partner (reference category), single, separated, divorced, widowed/widower), number of dependent children (continuous, 0–4), employment status (full-time with employer (reference category), self-employed, part-time with employer, not working (homemaker, full-time student, permanently unable to work, unemployed/temporarily laid off, and retired), annual household income (less than $25,000, $25,000 to $49,999, $50,000 to $74,999 (reference category), $75,000 to $99,999, $100,000 to $149,999, and $150,000 or more), education attainment (HS and less than HS (no high school diploma, high school diploma, high school diploma equivalent), some college, Associate’s degree, Bachelor’s degree (reference category), post-graduate studies) and military service (military service current member, previous member, never served (reference category)).

All questionnaire items are presented in S2 File.

### Empirical approaches

For descriptive data analysis, we use means and t-tests for means comparisons between respondents with and without alternative financial services, and Pearson’s r correlation analysis of alternative financial services use and the focal predictor variables. To address Research Questions 1, 2, and 3, we use binary logistic regression with a logit functional form, due to the binary nature of most of the outcome variables. This statistical approach is widely used for these data and type of specifications, and facilitates interpretation [46]. For robustness, due to the larger number of zeros in the outcome measures, probit regression analyses of the main specifications showed similar results, see S3 Table. For the specification that uses a count variable as the outcome measure, we use negative binomial regression [47].

All regression analyses follow the same three steps. In Step 1, we regress alternative financial services use on the scarcity mindset. In Step 2, we add established predictor variables of alternative financial services use, specifically objective and subjective financial knowledge, willingness to take financial risk, and difficulty covering monthly household expenses. In Step 3, for the full model, we regress alternative financial services use on the scarcity mindset, the established predictor variables, and sociodemographic controls.

### Descriptive statistics

Sample characteristics of individuals using alternative financial services in 2021 are shown in Tables 1 and 2, including the variables’ scale of measurement. Alternative financial services users in our sample were on average 40 years old, about 67% non-Hispanic White, and 48% were male. About 40% were married and less than half, 49% had dependent children. About 25% had a bachelor’s or post graduate degree. The largest represented annual income segment was less than $25,000, at about 31%. The majority of alternative financial services users worked full-time at close to 43%. The majority, or about 85%, of AFS users had never served in the U.S. Armed Services, see Table 1.

**Table 1:** Descriptive statistics of control variables, total and by alternative financial services use.

Control variables	Total sample	Alternative financial services user	Alternative financial services non-user
	% or Mean (SD)	% or Mean (SD)	% or Mean (SD)
			
Age (18–96)	47.89 (17.12)	39.97 (14.20)	52.03*** (16.78)
Male (0/1)	46.57%	47.58%	46.11%***
Race/Ethnicity			
White non-Hispanic (0/1)	74.84%	66.83%	78.55%***
Black non-Hispanic (0/1)	9.47%	15.75%	6.56%***
Hispanic (0/1)	8.20%	10.69%	7.05%***
Asia/Pacific Islander (0/1)	4.30%	2.69%	5.05%***
Other non-Hispanic (0/1)	3.19%	4.05%	2.79%***
Marital Status			
Married/Living with partner (0/1)	49.92%	39.97%	54.54%***
Single (0/1)	32.39%	42.53%	27.69%***
Separated (0/1)	1.72%	3.24%	1.01%***
Divorced (0/1)	11.44%	11.30%	11.51%***
Widowed/widower (0/1)	4.53%	2.96%	5.25%***
Number dependent children (0–4)	0.64 (1.039)	0.95 (1.89)	0.49*** (0.93)
Educational Attainment			
High School, equivalent or less (0/1)	25.83%	36.23%	21.02%***
Some college (0/1)	26.17%	28.51%	25.08%***
Associate’s degree (0/1)	11.01%	10.14%	11.42%**
Bachelor’s degree (0/1)	25.57%	17.75%	29.19%***
Postgraduate (0/1)	5.93%	7.37%	13.29%***
Employment Status			
Self-employed (0/1)	7.92%	9.98%	6.97%***
Work for employer full time (0/1)	39.61%	43.21%	37.94%***
Work for employer part time (0/1)	8.52%	10.16%	7.76%***
Not working (0/1)	43.95%	36.66%	47.33%***
Annual Income			
Less than $25,000 (0/1)	21.36%	31.42%	16.70%***
$25,000 to $49,999 (0/1)	24.93%	29.02%	23.03%***
$50,000 to $74,999 (0/1)	18.96%	15.61%	20.52%***
$75,000 to $99,999 (0/1)	13.52%	9.77%	15.26%***
$100,000 to $149,999 (0/1)	13.32%	9.37%	15.16%***
$150,000 or more (0/1)	7.91%	4.83%	9.33%***
Armed Services			
Current member (0/1)	2.13%	5.46%	0.58%***
Previous member (0/1)	10.40%	9.57%	10.78%**
Never member (0/1)	87.47%	84.97%	88.64%***

Notes: *** p < 0.001, ** p < 0.01, *p < .005, indicate means comparison results between respondents that used and did not use alternative financial services; N = 24,349

**Table 2:** Descriptive statistics of focal predictor variables, total and by alternative financial services use.

	Total sample	Alternative financial services user	Alternative financial services non-user
	% or Mean (SD)	% or Mean (SD)	% or Mean (SD)
Alternative financial services use (0/1)	31.66%		
Focal predictors:			
Scarcity mindset score (3–15, low to high)	8.78 (3.67)	10.58 (3.17)	7.95*** (3.60)
Objective financial knowledge (0–6, number correct)	3.10 (1.67)	2.40 (1.48)	3.42*** (1.66)
Subjective financial knowledge (1–7, low to high)	5.10 (1.32)	4.94 (1.47)	5.17*** (1.24)
Difficulty covering monthly expenses			
- Not at all difficult (0/1)	56.36%	31.50%	67.88%***
- Somewhat difficult (0/1)	33.37%	47.74%	26.72%***
- Very difficult (0/1)	10.27%	20.77%	5.40%***
Willingness to take financial risk (1–10, low to high)	5.08 (2.69)	5.58 (2.85)	4.84*** (2.58)

Notes: *** p < 0.001, ** p < 0.01, *p < .05, indicate means comparison results between respondents that use and do not use alternative financial services; N = 24,349

Individuals using alternative financial services differed from individuals not using alternative financial services with regard to all predictor variables at *p* < 0.001. Specifically, alternative financial services users report a higher scarcity score (M = 10.6, SD = 3.2) compared to non-users (M = 7.9, SD = 3.6); lower objective financial knowledge, with a mean score out of 6 of 2.4 (SD = 1.5) vs. 3.4 (SD = 1.7); lower subjective financial knowledge, with a mean score out of 7 of 4.9 (SD = 1.5) vs. 5.2 (SD = 1.2); greater willingness to take financial risk, with a mean score out of 10 of 5.6 (*SD* = 2.9) vs. 4.8 (*SD* = 2.6); difficulty covering monthly expenses with 31.5% of alternative financial services users reporting not at all difficult vs. 67.9% of non-users of alternative financial services, 47.7% of alternative financial services users reporting somewhat difficult vs. 26.7% of non-users of alternative financial services, and 20.8% of alternative financial services users reporting very difficult vs. 5.4% of non-users of alternative financial services, see Table 2.

The Pearson’s correlation coefficients of alternative financial services use, and the focal predictor variables is shown in Table 3. The correlation coefficient between alternative financial services uses and the scarcity mindset score is strong and positive at 0.332, p < 0.001. Alternative financial services use is inversely related with objective and subjective financial knowledge at −0.283 and −0.081, respectively, and is positively associated with willingness to take risk at 0.127 and difficulty covering monthly expenses at 0.357, p < 0.001.

**Table 3:** Correlation of alternative financial services use and focal predictor variables.

	Alternative financial services use	Scarcity mindset score	Objective financial knowledge	Subjective financial knowledge	Willingness to take financial risk
	Pearson’s r	Pearson’s r	Pearson’s r	Pearson’s r	Pearson’s r
Scarcity mindset score	0.332***				
Objective financial knowledge	−0.283***	−0.295***			
Subjective financial knowledge	−0.081***	−0.313***	0.255***		
Willingness to take financial risk	0.127***	−0.087***	0.118***	0.282	
Difficulty covering monthly expenses	0.357***	0.612***	−0.259***	−0.250***	−.070***

Notes: ***p < 0.001 **p < 0.01 *p < 0.05; N = 24,349

## Results

### Scarcity mindset’s association with use of alternative financial services

To examine Research Questions 1 and 2, the association of alternative financial services use and scarcity mindset. Results are reported in Table 4 as odds ratios. The results of Step 1, the reduced-form specification for Research Question 1, indicate a strong and positive association of alternative financial services use and the scarcity mindset. For individuals with a one-unit stronger scarcity mindset, the odds of using alternative financial services are higher by a factor of 1.235 or 23.5%.

**Table 4:** Logistic regression of alternative financial services use on scarcity mindset.

Variable	(1) Use of any alternative financial services	(2) Use of any alternative financial services	(3) Use of any alternative financial services
	OR (SE)	OR (SE)	OR (SE)
Scarcity mindset	1.235*** (0.005)	1.121*** (0.006)	1.100*** (0.007)
Established predictors:			
Objective financial knowledge		0.722*** (0.007)	0.811*** (0.009)
Subjective financial knowledge		1.049*** (0.013)	1.087*** (0.015)
Willingness to take financial risk		1.184*** (0.007)	1.099*** (0.008)
Difficulty covering monthly expenses (Ref.: Not at all difficult)			
Somewhat difficult		2.473*** (0.095)	2.052*** (0.084)
Very difficult		4.228*** (0.242)	3.245*** (0.198)
Demographic controls:			
Age			0.967*** (0.001)
Male			1.281*** (0.047)
Race/Ethnicity (Ref.: White non-Hispanic)			
Black non-Hispanic			1.814*** (0.099)
Hispanic (alone/comb)			1.120* (0.063)
Asia/Pacific Islander			0.722*** (0.066)
Other non-Hispanic			1.263** (0.111)
Marital Status (Ref.: Married)			
Single			0.835*** (0.038)
Separated			2.025*** (0.242)
Divorced			1.319*** (0.075)
Widowed/widower			1.411*** (0.131)
Dependent Children			1.276*** (0.021)
Educational Attainment (Ref.: Bachelor’s degree)			
High School, equivalent or less			1.694***(0.085)
Some college			1.427*** (0.069)
Associate’s degree			1.221** (0.075)
Postgraduate			1.004 (0.068)
Employment Status (Ref.: full time)			
Self-employed			1.060 (0.066)
Work for employer part time			0.941 (0.057)
Not working			0.839*** (0.036)
Annual Income (Ref.: $50,000-$74,999)			
Less than $25,000			1.333*** (0.075)
$25,000 to $49,999			1.260*** (0.064)
$75,000 to $99,999			0.917 (0.057)
$100,000 to $149,999			0.952 (0.062)
$150,000 or more			0.926 (0.077)
Armed Services (Ref.: Never member)			
Current member			3.488*** (0.449)
Previous member			1.539*** (0.090)
Constant	0.065*** (0.003)	0.080*** (0.007)	0.650*** (0.094)
Log likelihood	−13,803.180	−12,437.118	−11,425.446
Pseudo R^2^	0.092	0.182	0.248

Notes: N = 24,349; ***p < 0.001 **p < 0.01 *p < 0.05

For Research Question 2, we find that the association of alternative financial services use, and scarcity mindset is robust to accounting for established predictors in Model 2, and to accounting for socioeconomic controls in Model 3. In Model 3, the scarcity mindset score remains significant at *p* < 0.001. An odds ratio of 1.100 implies that a one-unit stronger scarcity mindset is associated with 10.0% higher odds of using alternative financial services. The strong relationship is noteworthy because is it robust to a set of four established predictors that are strongly associated with higher odds of alternative financial services use by 8.7% for a one-point higher subjective financial knowledge score, by 9.9% for a one-point higher willingness to take financial risk, and by 325% for high difficulty covering monthly expenses, compared to no difficulty. The odds ratio of alternative financial services use is 0.811 or lower by 18.9% for a one-point higher objective financial knowledge score. Testing for multicollinearity with the Variance Inflation Factor (VIF) shows a negligible amount in Model 2, with a mean VIF of 1.37 and a VIF range of 1.09 to 1.76, and also in Model 3 with a mean VIF of 1.42 and a VIF range of 1.02 to 2.17.

### Scarcity mindset’s association with count and type of alternative financial services

**Fig 1:**
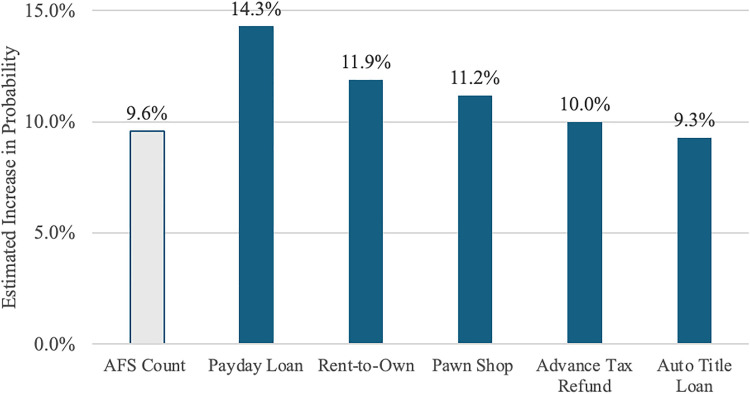
Estimated increase in the probability of number and type of alternative financial services on scarcity mindset. Negative binomial regression model for AFS count; binary logistic regression models for payday loans, rent-to-own stores, pawn shops, advance on tax refund, auto title loans.

We further examine the association of a scarcity mindset with the count of individuals’ use of alternative financial services and with each of the five alternative financial services, including payday loans, rent-to-own stores, pawn shops, advance on tax refunds, and auto title loans (Fig 1, S4 Table). Results of negative binomial regression show that a one-unit higher level of scarcity mindset is associated with using 9.6 percentage points more alternative financial services. Results of binary logistic regression document a small but significant positive relationship with each type, with a one-unit increase of scarcity mindset associated with a 14.3 percentage point higher use of payday loans, 11.9 percentage point higher use of rent-to-own, 11.2 percentage point higher use of pawn shops, 10.0 percentage point higher use of advance tax refunds, and 9.3 percentage point higher use of auto title loans.

### Scarcity mindsets’ association with the use of alternative financial services by household income

For Research Question 3, the analytical sample was divided into three subsamples of low-income respondents, or less than $50,000 annual household income, middle-income respondents, or $50,000 to less than $100,000 annual income, and high-income respondents, of $100,000 and more annual household income. We stratified the 10 income bands used in the 2021 survey into three income groups: low-income respondents (less than $50,000 annual household income), middle-income respondents ($50,000 to less than $100,000 annual income), and high-income respondents ($100,000 or greater annual household income). This approach was informed by estimates on income, earnings, and inequality in the United States for calendar year 2021 of the U.S. Census Bureau [48]. This analysis tests whether the association of alternative financial services use and scarcity mindset differs across income groups because financial vulnerability has been the focus of much of the research on alternative financial services use [8].

**Fig 2:**
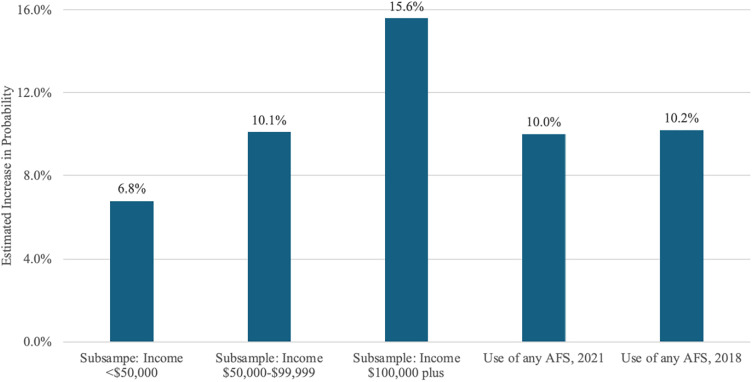
Estimated increase in the probability of using alternative financial services by household income and survey year.

Results for the binary logistics regression are shown in Table 5. We find in each subsample a significant association of the scarcity mindset with alternative financial services use at p < 0.001 with slightly higher odds for higher household incomes (Fig 2). For individuals with a one-unit stronger scarcity mindset, the odds of using alternative financial services are higher by a factor of 1.068 or 6.8% for household income of less than $50,000, 1.101 or 10.1% for household income of %50,000 to $99,999, and 1.156 or 15.6% for household income of $100,000 and higher. Although the high-income respondents are lesser users of alternative financial services – 21.2% use alternative financial services in our sample – the scarcity mindset transcends income status and emerges as a driver of alternative financial services use among wealthier households.

**Table 5:** Logistic regression of alternative financial services use on scarcity mindset, by household income subsamples.

Variable	(1) Household income less than $50,000	(2) Household income $50,000 to $99,999	(3) Household income $100,000 or more
	OR (SE)	OR (SE)	OR (SE)
Scarcity mindset	1.068*** (0.009)	1.101*** (0.012)	1.156*** (0.018)
Objective financial knowledge	0.900*** (0.014)	0.788*** (0.017)	0.658*** (0.020)
Subjective financial knowledge	1.050** (0.017)	1.064* (0.029)	1.211*** (0.054)
Willingness to take financial risk	1.090*** (0.010)	1.098*** (0.015)	1.120*** (0.024)
Difficulty covering monthly expenses (Ref.: Not at all difficult)			
Somewhat difficult	2.024*** (0.112)	2.229*** (0.163)	1.831*** (0.202)
Very difficult	3.259*** (0.241)	3.433*** (0.484)	3.353*** (0.855)
Demographic controls	YES	YES	YES
Log likelihood	−6194.925	−3355.951	−1734.438
Pseudo R2	0.189	0.242	0.350
N (subsample)	11,270	7,910	5,169
% of DV	41.3%	24.7%	21.2%
N of subsample	11,270	7,910	5,169

Notes: N = 24,349; ***p < 0.001 **p < 0.01 *p < 0.05; Demographic controls include age, race/ethnic identity, gender, marital status, child dependency status, educational attainment, income, work status, and US armed services experience.

### Robustness: Scarcity mindset and its association with the use of alternative financial services in pre-COVID 2018

We repeat the analyses with the pre-COVID 2018 survey year to assess the robustness of the findings for the 2021 survey year. Descriptive statistics for 2018 are shown in S5 and S6 Table. The data underlying the results presented in the study are available from the FINRA Investor Education Foundation [43]. Use of alternative financial services in 2018, at 27.4%, was lower as a percentage of the sample survey than in 2021, at 31.7%, based on these samples which were stratified by age, gender, ethnicity, education level, and income. Results for the binary logistics regression show a strong and positive association of alternative financial services use and scarcity mindset, see S7 Table. The coefficients are similar to the 2021 results, indicating that a one-unit stronger scarcity mindset is associated with 10.2% higher odds of using alternative financial services (Fig 2). The association of established predictors with alternative financial services use is stable and reflects the same directions and similar magnitudes in the 2018 and 2021 data.

## Discussion

This study uses a behavioral economic framework – the scarcity mindset framework – to contribute new insights into the mechanisms underlying alternative financial services use, reflecting risky consumer financial decision-making. The study uses recent survey data from the 2021, and then by extension 2018, National Financial Capability Study State-by-State Survey. Interpretation of results show that a stronger scarcity mindset is significantly and positively related to alternative financial services use across all specifications and controlling for established predictors of alternative financial services use and socio-demographic characteristics. Specifically, our findings document that, for individuals with a one-unit stronger scarcity mindset, the odds of using alternative financial services are higher by about 10% in both the 2021 and 2018 data sets. At 14.3%, the odds are higher for the use of payday loans and, at 15.6%, among those with higher household incomes. The findings suggest that the scarcity mindset provides a unique, direct, and small but meaningful explanation for consumers using alternative financial services, above and beyond established predictors and a rich set of demographic factors.

### Theoretical and policy interpretation and directions for future research

The unadjusted results from our main specification show that the scarcity mindset has significant explanatory power in consumer financial decision-making which aligns with the initial research on the economic framework that underlies the scarcity mindset [5]. Interpretation of this finding suggests the need for further research into the connection between the scarcity mindset and a wider range of financial decision-making and service/product selection. The results from our full specifications document the explanatory power of the scarcity mindset in consumer financial decision-making, above known predictors and sociodemographic factors such as financial literacy [35], willingness to take financial risk [34], income and objective financial scarcity [13]. The significant role of the scarcity mindset in the current study suggests that a continued focus on measurement of the scarcity mindset and related underlying psychological mechanisms is a worthwhile and specific direction for future research.

The analytical extensions in the number of alternative financial services used by an individual and the types of financial services used corroborate other research into the characteristics and motivations of alternative financial services users [49–51]. As the proliferation of alternative financial services increases [52, 53], research could examine the nuanced connection between the scarcity mindset and the mechanisms that drive the selection of one alternative financial service over another and that drive the frequency of usage. As our results show, the popularity of certain alternative financial services do not necessarily align with their relationship to the scarcity mindset. For example, while the scarcity mindset predicted the highest engagement with payday lending (Odds Ratio: 1.143), payday lending was not the most widely used alternative financial service (12.2% vs 20.2% for advance tax refunds).

The consistency of the results for the 2021 and pre-COVID 2018 survey data further suggests that the scarcity mindset might be a stable personal characteristic, as suggested in the context of decision-making among low-income individuals [13, 14]. Specific directions for future research include an examination of the temporal, contextual, and stable psychological factors that help explain a person’s psychological make-up that contributes to financial decision-making.

The segmentation into low-, middle-, and high-income subsamples corroborates initial research into the role of perceived scarcity (i.e., subjective scarcity) and income in financial decision-making [54], and impressively underscores the scarcity mindset’s explanatory power. These results, in particular, justify a stronger focus on the psychological mechanisms that may underly the scarcity mindset and may explain poor or mistaken financial decisions.

### Practical recommendations relevant to practitioners or service providers

The study findings have practical implications for regulators, policymakers, financial service providers, and researchers. First, study findings relate to measurement of the scarcity mindset with regard to survey instruments to enhance the measurement of the scarcity mindset. The current study tests a new approach by using an existing instrument that can inform future surveys and experiments, adding tools to the existing Psychological Inventory of Financial Scarcity [25], the Perceived Scarcity Scale [27], and the Perceived Economic Scarcity Scale [28]. Judging from the current study, which used survey questions, these instruments are useful in teasing apart nuances in subjective and objective scarcity underlying the scarcity mindset. For example, the Psychological Inventory of Financial Scarcity has been used to develop a four-factor model, measuring shortage of money, lack of control, rumination and worry, and short-term focus. Based on the current study, these four factors could improve the process of better understanding the scarcity mindset [25]. Furthermore, using one of the newly designed scarcity mindset measurement instruments in conjunction with well-designed experiments could allow for analysis of the connection between the key features of the scarcity mindset – namely, the bandwidth tax, tunneling, and trade-off thinking – and financial decision-making [5].

The ongoing development of alternative financial services provides continued reasons for focusing on these risky financial tools. First, starting in 1994, Caskey [55] documented the use of fringe banking services – pawnshops and check-cashing outlets – especially on those in poverty, and subsequently he documented and analyzed the slowdown in pawn shop activities in concert with the rise in payday lending. Second and more recently, another alternative financial service has grown in popularity: the buy now, pay later product [56]. Demographically, there are similarities with users of other alternative financial services with the dominant buy now, pay later users being 18–35 years of age, incomes of more than $70,000, of Black race, or Hispanic ethnicity for buy now, pay later products. Despite the finding that users of buy now, pay later products tend to make payments on time, compared to non-users they were more likely to have experienced financial disruption, difficulty covering monthly expenses, and difficulty to cover a $400 expense [56]. Third, the growing popularity of earned wage advances, which are payday loan-like mobile apps that allow an advance on a paycheck, documents users’ willingness to pay exorbitant average annual percentage rates [52]. Fourth, consumer data have documented the significant positive relationship between ownership of and investment in cryptocurrency and purchase of a money orders from an institution other than a bank and payment of an overdraft fee on a bank account [57]. User profiles of these newer financial products align with the current study sample, and our findings may suggest the scarcity mindset as a potential explanatory factor.

For example, further research might examine the extent to which use of cryptocurrency as an alternative financial service could place vulnerable people at risk given the paucity of regulation of cryptocurrency.

In addition to new alternative financial services products, there is a blurring of lines with the expansion of financial services platforms with fintech and cultural phenomena, in what has been described as the gamification of finance [58]. The easy availability of financial trading and gambling apps on smartphones, combined with the perception of not having enough compared to peers makes another case for assessing the role of the scarcity mindset for fintech-based financial decision-making and gambling [58].

The positive association between a scarcity mindset and the use of alternative financial services, such as payday loans and pawn shops, should be of concern among regulators, policymakers, and government and business leaders. A useful direction for future research is to investigate the connection between and overlap with the scarcity mindset psychological constructs related to materialism, specifically fear of missing out (FOMO) which reflects persons’ feelings of anxiety or apprehension that they are missing out on what others are enjoying [59, 60], you only live once (YOLO) which is a psychological construct where an individual makes short term decisions with limited regard to future consequences and risks [61, 62], and financial nihilism, which is a term coined to represent a mindset that the driver in trading and investing is the narrative and emotion around the asset rather than its value [63]. The scarcity mindset should be examined within the context of these psychological factors and mechanisms that motivate individuals to use alternative financial services.

### Limitations

Study limitations should be noted. First, the survey questions about alternative financial services use inquired about activities over the past 5 years, but the scarcity questions were contemporaneous to the date of the data collection. The different time frames of the questions may bias the analyses. However, studies have shown that survey respondents prioritize current events over past events, indicating a bias toward the present. As a result, the survey question about alternative financial services use in the National Financial Capability study has been used in a number of studies in the way we use it in the current study [31, 36, 40]. Second, our measures of the scarcity mindset and objective financial scarcity, here measured by difficulties in covering monthly expenses, are positively correlated. This result supports the notion forwarded by Auger, Sommet and Normand [54] that a scarcity mindset and objective financial scarcity are positively and significantly related. Future research should explore the relative role of a scarcity mindset vs objective monetary scarcity as mechanisms for financial decision-making. This future research could mirror research on the role of subjective and objective financial knowledge [27, 64].

## Conclusion

Managing scarcity, and rational actions in this process, has long been central to economics [65]. Scarcity mindset theory expanded the field of behavioral economics by naming certain attributes that can be identified among individuals that do not have what they feel that they need expanding the notion of scarcity beyond tangible, objective needs; these attributes include a cognitive tax on mental bandwidth, tunneling in decision-making, and mistaken and/or suboptimal decisions [5]. In this paper, we presented evidence of an association between the presence of a scarcity mindset and negative financial outcomes, namely the use of alternative financial services, such as payday loans, rent-to-own stores, pawn shops, advance on tax refunds, and auto title loans.

## Supporting information

S1 File Protocol of expert survey for development of scarcity mindset measure. (DOCX)

S2 File Survey questionnaire. (DOCX)

S3 Table Coefficients of probit regression of alternative financial services use on scarcity mindset. (DOCX)

S4 Table Coefficients of regression of count and type of alternative financial services use on scarcity mindset. (DOCX)

S5 Table Descriptive statistics of focal predictor variables, 2018 data collection. (DOCX)

S6 Table Descriptive statistics of control variables, 2018 data collection. (DOCX)

S7 Table Logistic regression of alternative financial services use on scarcity mindset, 2018 data collection. (DOCX)
