# Supplementary material for: Scarcity mindset’s positive association with using alternative financial services
Source: PLoS One. 2026 Feb 20;21(2):e0339127. doi: 10.1371/journal.pone.0339127 (PMC12923054; doi:10.1371/journal.pone.0339127)
Supplement: S1 File — (DOCX) [file pone.0339127.s001.docx]

**S1 File. Protocol of Expert Survey for Development of Scarcity Mindset Measure.**

*Note: The information provided in this S2 Appendix is identical to information provided in Rooney G, Loibl C. Scarcity mindset as predictor of high-risk investments. Social Sciences Research Network. 2025;5157305. doi: 10.2139/ssrn.5157305*

Experts:

A group of 7 researchers with academic publications on the topic of scarcity mindset were contacted on March 1, 2023 and invited to provide feedback on the development of the scarcity mindset variable. The group consisted of 5 economists and 2 psychologists. They agreed to participate based on earlier email inquiries. After receiving the instructions, each expert responded with their rating within 1 to 2 weeks in writing.

Written instructions to experts:

In the book *Scarcity: The new science of having less and how it defines our lives*, Mullainathan and Shafir (2013) put forth the framework that scarcity is a mindset that can be summarized as “having less than you feel you need”. According to the authors, scarcity captures attention and “changes the way we think” and behave.

The scarcity-mindset framework has particular attributes:

- Tunnelling (defined as “singlemindedness” that can lead to neglect)
- “Bandwidth tax” (defined as limitations to “cognitive capacity” and “executive control”)
- Myopia (defined as neglecting planning and the future)

In the absence of a survey instrument that measures an individual’s scarcity mindset, we propose to use specific questions from FINRA’s National Financial Capability Study as proxies to measure the “scarcity mindset”. In the table, I list specific questions that I selected to measure the scarcity mindset. We would like to ask you to rate and comment on their potential fit.

Expert responses to proposed survey questions (* indicates chosen measures):

Survey question 1: Overall, thinking about your assets, debts and savings, how satisfied are you with your current personal financial condition?

Response options: 1 Not at all satisfied to 10 Extremely satisfied

Survey question 2: How often do you think about your personal financial condition?

Response options: 1 Never to 6 More than once a day

*Survey question 3: Because of my money situation, I feel like I will never have the things I want in life

Response options: 1 Does not describe me at all to 5 Defines me completely

*Survey question 4: I am concerned that the money I have or will save won’t last.

Response options: 1 Does not describe me at all to 5 Defines me completely

*Survey question 5: I am just getting by financially.

Response options: 1 Does not describe me at all to 5 Defines me completely

Survey question 6: I have too much debt right now.

Response options: 1 Strongly agree to 7 strongly disagree

Survey question 7: Are you concerned that you might not be able to pay off your student loans?

Response options: Yes or No

Survey question 8: Do you currently owe more on your home than you think you could sell it for today?

Response options: Yes/owe more, or No

Survey question 9: If you were to set a financial goal for yourself for today, how confident are you in your ability to achieve it?

Response options: 1 Not all confident to 4 Very confident

Survey question 10: How confident are you that you could come up with $2,000 if an unexpected need arose within the next month?

Response options: 1 I am certain I could come up with the full $2,000 to 4 I am certain I could not come up with $2,000

Expert rating:

Experts 1 to 5: Economists

Experts 6, 7: Psychologists

| Survey question | Expert 1 | Expert 2 | Expert 3 | Expert 4 | Expert 5 | Expert 6 | Expert 7 |
| --- | --- | --- | --- | --- | --- | --- | --- |
| 1 | No | No | Maybe | Yes | No | Yes | Maybe |
| 2 | No | No | Maybe | Maybe | Maybe | Yes | Maybe |
| 3-chosen | Yes | Yes | Maybe | Yes | Yes | Yes | Maybe |
| 4-chosen | Yes | Yes | Maybe | Yes | Yes | Yes | Maybe |
| 5-chosen | Yes | Yes | Maybe | Yes | Yes | Yes | Maybe |
| 6 | No | No | Maybe | Yes | Yes | Maybe | Maybe |
| 7 | No | Yes | Maybe | Maybe | No | Maybe | Maybe |
| 8 | No | No | Maybe | Maybe | No | Maybe | Maybe |
| 9 | No | Yes | Maybe | Maybe | No | No | Maybe |
| 10 | No | Yes | Maybe | Yes | No | Maybe | Maybe |

Survey questions 3, 4, and 5 received 5 yes ratings out of 7 ratings and were chosen to comprise the scarcity mindset measure. Reliability: Cronbach’s alpha 0.87 in 2021 and 0.86 in 2018
