# Supplementary material for: Scarcity mindset’s positive association with using alternative financial services
Source: PLoS One. 2026 Feb 20;21(2):e0339127. doi: 10.1371/journal.pone.0339127 (PMC12923054; doi:10.1371/journal.pone.0339127)
Supplement: S2 File — (DOCX) [file pone.0339127.s002.docx]

# S2 File. Survey Questionnaire.

Alternative financial services questions

In the past 5 years, how many times have you ….

- Taken out an auto title loan? Auto title loans are loans where a car title is used to borrow money for a short period of time. They are NOT loans used to purchase an automobile.
- Taken out a short term ‘payday’ loan?
- Gotten an advance on your tax refund? This is sometimes called a ‘refund anticipation check’ or ‘Rapid Refund’ (Not the same as e-filing)
- Used a pawn shop?
- Used a rent-to-own store?

Response options:

- Never
- 1 time
- 2 times
- 3 times
- 4 or more times
- Don’t know
- Prefer not to say

Scarcity mindset

How well do these statements describe you or your situation?

- Because of my money situation, I feel like I will never have the things I want in life.
- I am just getting by financially.
- I am concerned that the money I have or will save won’t last.

Response options:

- Does not describe me at all
- Describes me very little
- Describes me somewhat
- Describes me very well
- Describes me completely
- Don’t know
- Prefer not to say

Financial literacy

Suppose you had $100 in a savings account and the interest rate was 2% per year. After 5 years, how much do you think you would have in the account if you left the money to grow?

Response options:

- More than $102
- Exactly $102
- Less than $102
- Don’t know
- Prefer not to say

Imagine that the interest rate on your savings account was 1% per year and inflation was 2% per year. After 1 year, how much would you be able to buy with the money in this account?

Response options:

- More than today
- Exactly the same
- Less than today
- Don’t know
- Prefer not to say

If interest rates rise, what will typically happen to bond prices?

Response options:

- They will rise
- They will fall
- They will stay the same
- There is no relationship between bond prices and the interest rate
- Don’t know
- Prefer not to say

Suppose you owe $1,000 on a loan and interest rate you are charged is 20% per year compounded annually. If you didn’t pay anything off, at this interest rate, how many years would it take for the amount you owe to double?

Response options:

- Less than 2 years
- At least 2 years but less than 5 years
- At least 5 years but less than 10 years
- At least 10 years
- Don’t know
- Prefer not to say

A 15-year mortgage typically requires higher monthly payments than a 30-year mortgage, but the total interest paid over the life of the loan will be less.

Response options:

- True
- False
- Don’t know
- Prefer not to say

Buying a single company’s stock usually provides a safer return than a stock mutual fund.

Response options:

- True
- False
- Don’t know
- Prefer not to say

Subjective financial knowledge

On a scale from 1 to 7, where 1 means very low and 7 means very high, how would you assess your overall financial knowledge?

- Responses on 7-point scale, 1=very low to 7=very high
- Don’t know
- Prefer not to say

Willingness to take financial risk

When thinking about financial investments, how willing are you to take risks?

- Responses on 10-point scale, 1=Not At All Willing to 10=Very Willing
- Don’t know
- Prefer not to say

Difficulty covering monthly expenses

In a typical month, how difficult is it for you to cover your expenses and pay all your bills?

Response options:

- Very difficult
- Somewhat difficult
- Not at all difficult
- Don’t know
- Prefer not to say
