# Supplementary material for: Scarcity mindset’s positive association with using alternative financial services
Source: PLoS One. 2026 Feb 20;21(2):e0339127. doi: 10.1371/journal.pone.0339127 (PMC12923054; doi:10.1371/journal.pone.0339127)
Supplement: S3 Table — (DOCX) [file pone.0339127.s003.docx]

**S3 Table. Coefficients of Probit Regression of Alternative Financial Services Use on Scarcity Mindset.**

| Variable | (1) Use of any alternative financial services | (2) Use of any alternative financial services | (3) Use of any alternative financial services |
| --- | --- | --- | --- |
|  | OR (SE) | OR (SE) | OR (SE) |
| Scarcity mindset | 0.128*** (0.002) | 0.068*** (0.003) | 0.055*** (0.003) |
| Objective financial knowledge |  | -0.193*** (0.006) | -0.123*** (0.007) |
| Subjective financial knowledge |  | 0.028*** (0.007) | 0.049*** (0.008) |
| Willingness to take financial risk |  | 0.098*** (0.004) | 0.054*** (0.004) |
| Difficulty covering monthly expenses (Ref.: Not at all difficult) |  |  |  |
| Somewhat difficult |  | 0.534*** (0.023) | 0.424*** (0.024) |
| Very difficult |  | 0.859*** (0.034) | 0.699*** (0.036) |
| Demographic controls | NO | NO | YES |
| Log likelihood | -13788.643 | -12422.404 | -11420.467 |
| Pseudo R2 | 0.093 | 0.183 | 0.249 |

Notes: ***p<0.001 **p< 0.01 *p<0.05; N=24,349

Demographic controls: Age, gender, race/ethnic identity, marital status, dependent children, educational attainment, employment status, annual income, armed services
