# Supplementary material for: Scarcity mindset’s positive association with using alternative financial services
Source: PLoS One. 2026 Feb 20;21(2):e0339127. doi: 10.1371/journal.pone.0339127 (PMC12923054; doi:10.1371/journal.pone.0339127)
Supplement: S4 Table — (DOCX) [file pone.0339127.s004.docx]

**S4 Table. Coefficients of Regression of Count and Type of Alternative Financial Services Use on Scarcity Mindset.**

| Predictor  Variable | (1) AFS Count | (2) Payday Loan | (3) Rent-to-Own | (4) Pawn Shop | (5) Advance Tax Refund | (6) Auto Title Loan |
| --- | --- | --- | --- | --- | --- | --- |
|  | IRR (SE) | OR (SE) | OR (SE) | OR (SE) | OR (SE) | OR (SE) |
| Scarcity mindset | 1.096*** (0.005) | 1.143*** (0.009) | 1.119*** (0.009) | 1.112*** (0.008) | 1.100*** (0.010) | 1.093*** (0.009) |
| Objective financial knowledge | 0.837*** (0.007) | 0.832*** (0.012) | 0.786*** (0.012) | 0.833*** (0.011) | 0.748*** (0.013) | 0.798*** (0.012) |
| Subjective financial knowledge | 1.080*** (0.010) | 1.135*** (0.029) | 1.150*** (0.020) | 1.089*** (0.016) | 1.249*** (0.026) | 1.184*** (0.022) |
| Willingness to take financial risk | 1.088*** (0.005) | 1.118*** (0.010) | 1.100*** (0.010) | 1.116*** (0.009) | 1.150*** (0.012) | 1.119*** (0.010) |
| Difficulty covering monthly expenses (Ref.: Not at all difficult) |  |  |  |  |  |  |
| Somewhat difficult | 1.708*** (0.050) | 2.0416*** (0.108) | 1.803*** (0.100) | 2.005*** (0.096) | 1.832*** (0.114) | 1.620*** (0.091) |
| Very difficult | 2.102*** (0.085) | 2.977*** (0.213) | 2.100*** (0.157) | 3.333*** (0.217) | 2.211*** (0.191) | 1.987*** (0.155) |
| Demographic controls | YES | YES | YES | YES | YES | YES |
| Log likelihood | -21554 | -7708 | -7123 | -8954 | -5570 | -6984 |
| Pseudo R2 | 0.146 | 0.232 | 0.228 | 0.251 | 0.294 | 0.205 |
| N | 23,607 | 24,098 | 24,122 | 24,137 | 23,985 | 24,118 |
| % or Mean of DV (SD) | 0.678 (1.309) | 12.2% | 14.9% | 10.7% | 20.2% | 13.2% |
| Range | 0-5 | 0/1 | 0/1 | 0/1 | 0/1 | 0/1 |

Notes: ***p<0.001 **p< 0.01 *p<0.05

Demographic controls: Age, race/ethnic identity, gender, marital status, child dependency status, educational attainment, income, work status, and US armed services experience;

Model (1) uses negative binomial regression; Models (2)-(5) use binary logistic regression
