# Supplementary material for: Scarcity mindset’s positive association with using alternative financial services
Source: PLoS One. 2026 Feb 20;21(2):e0339127. doi: 10.1371/journal.pone.0339127 (PMC12923054; doi:10.1371/journal.pone.0339127)
Supplement: S5 Table — (DOCX) [file pone.0339127.s005.docx]

**S5 Table**. **Descriptive Statistics of Focal Predictor Variables, 2018 Data Collection.**

|  | Total sample | Alternative financial services user | Alternative financial services non-user |
| --- | --- | --- | --- |
|  | % or Mean (SD) | % or Mean (SD) | % or Mean (SD) |
| Alternative financial services use (0/1) | 27.37% |  |  |
| Focal predictors: |  |  |  |
| Scarcity mindset score (3-15) | 8.79 (3.69) | 10.69 (3.26) | 8.08*** (3.58) |
| Objective financial knowledge (0-6) | 3.26 (1.63) | 2.55 (1.46) | 3.53*** (1.62) |
| Subjective financial knowledge (1-7) | 5.17 (1.32) | 4.96 (1.52) | 5.25*** (1.22) |
| Difficulty covering monthly expenses |  |  |  |
| - Not at all difficult (0/1) | 54.11% | 29.67% | 63.31%*** |
| - Somewhat difficult (0/1) | 34.68% | 46.91% | 30.08%*** |
| - Very difficult (0/1) | 11.21% | 23.42% | 6.61%*** |
| Willingness to take financial risk (1-10) | 4.95 (2.65) | 5.36 (2.95) | 4.80*** (2.51) |

Notes: *** p<0.001, ** p<0.01, *p<.005, indicate means comparison results between respondents that use and do not use alternative financial services; N = 24,103
