# Supplementary material for: Scarcity mindset’s positive association with using alternative financial services
Source: PLoS One. 2026 Feb 20;21(2):e0339127. doi: 10.1371/journal.pone.0339127 (PMC12923054; doi:10.1371/journal.pone.0339127)
Supplement: S6 Table — (DOCX) [file pone.0339127.s006.docx]

**S6 Table**. **Descriptive Statistics of Control Variables, 2018 Data Collection.**

|  | Total sample | Alternative financial services user | Alternative financial services non-user |
| --- | --- | --- | --- |
|  | % or Mean (SD) | % or Mean (SD) | % or Mean (SD) |
| Control variables: |  |  |  |
| Age (18-92) | 48.13 (16.75) | 39.48 (14.07) | 51.39*** (16.51) |
| Male (0/1) | 44.82% | 45.62% | 44.51% |
| Race/Ethnicity |  |  |  |
| White non-Hispanic (0/1) | 75.15% | 62.48% | 79.93%*** |
| Black non-Hispanic (0/1) | 9.07% | 17.31% | 5.96%*** |
| Hispanic (0/1) | 8.38% | 12.02% | 7.01%*** |
| Asia/Pacific Islander (0/1) | 4.33% | 4.38% | 4.31% |
| Other non-Hispanic (0/1) | 3.07% | 3.81% | 2.79%*** |
| Marital Status |  |  |  |
| Married/Living with partner | 54.42% | 45.06% | 57.94%*** |
| Single | 28.59% | 38.05% | 25.02%*** |
| Separated | 1.46% | 2.47% | 1.09%*** |
| Divorced | 11.21% | 11.40% | 11.13% |
| Widowed/widower | 4.33% | 3.02% | 4.82%*** |
| Number dependent children (0-4) | 0.66 (1.05) | 1.02 (1.22) | 0.52*** (0.95) |
| Educational Attainment |  |  |  |
| High School, equivalent or less (0/1) | 25.98% | 34.20% | 22.88%*** |
| Some college (0/1) | 26.83% | 32.06% | 24.86%*** |
| Associate’s degree (0/1) | 10.70% | 10.76% | 10.68% |
| Bachelor’s degree (0/1) | 22.62% | 15.65% | 25.25%*** |
| Postgraduate (0/1) | 13.87% | 7.32% | 16.33%*** |
| Employment Status |  |  |  |
| Self-employed (0/1) | 7.37% | 8.61% | 6.91%*** |
| Work for employer full time (0/1) | 41.02% | 44.45% | 39.73%*** |
| Work for employer part time (0/1) | 8.62% | 10.14% | 8.04%*** |
| Not working (0/1) | 42.99% | 36.80% | 45.32%*** |
| Annual Income |  |  |  |
| Less than $25,000 (0/1) | 19.55% | 29.06% | 15.97%*** |
| $25,000 to $49,999 (0/1) | 25.12% | 30.52% | 23.08%*** |
| $50,000 to $74,999 (0/1) | 19.92% | 17.07% | 21.00%*** |
| $75,000 to $99,999 (0/1) | 14.80% | 13.16% | 15.42%*** |
| $100,000 to $149,999 (0/1) | 13.40% | 7.60% | 15.58%*** |
| $150,000 or more (0/1) | 6.36% | 2.59% | 8.96%*** |
| Armed Services |  |  |  |
| Current member (0/1) | 2.80% | 8.38% | 0.70%*** |
| Previous member (0/1) | 11.75% | 10.82% | 12.10%** |
| Never member (0/1) | 85.45% | 80.79% | 87.21%*** |

Notes: *** p<0.001, ** p<0.01, *p<.005, indicate means comparison results between respondents that use and do not use alternative financial services; N = 24,103
