# Supplementary material for: Scarcity mindset’s positive association with using alternative financial services
Source: PLoS One. 2026 Feb 20;21(2):e0339127. doi: 10.1371/journal.pone.0339127 (PMC12923054; doi:10.1371/journal.pone.0339127)
Supplement: S7 Table — (DOCX) [file pone.0339127.s007.docx]

**S7 Table**. **Logistic Regression of Alternative Financial Services Use on Scarcity Mindset, 2018 Data Collection.**

| Variable | (1) Use of any alternative financial services, 2018 | (1) Use of any alternative financial services, 2018 |
| --- | --- | --- |
|  | OR (SE) | OR (SE) |
| Scarcity mindset | 1.230*** (0.005) | 1.102*** (0.007) |
| Objective financial knowledge |  | 0.833*** (0.010) |
| Subjective financial knowledge |  | 1.029* (0.014) |
| Willingness to take financial risk |  | 1.105*** (0.008) |
| Difficulty covering monthly expenses  (Ref.: Not at all difficult) |  |  |
| Somewhat difficult |  | 1.709*** (0.073) |
| Very difficult |  | 2.420*** (0.149) |
| Demographic controls: |  |  |
| Age |  | 0.968*** (0.001) |
| Male |  | 1.2725*** (0.049) |
| Race/Ethnicity (Ref.: White non-Hispanic) |  |  |
| Black non-Hispanic |  | 2.159*** (0.121) |
| Hispanic (alone/comb) |  | 1.331*** (0.075) |
| Asia/Pacific Islander |  | 1.198* (0.010) |
| Other non-Hispanic |  | 1.305** (0.118) |
| Marital Status (Ref.: Married) |  |  |
| Single |  | 0.844*** (0.039) |
| Separated |  | 1.557** (0.198) |
| Divorced |  | 1.361*** (0.079) |
| Widowed/widower |  | 1.419*** (0.136) |
| Dependent Children |  | 1.266*** (0.021) |
| Educational Attainment (Ref.: Bachelor’s degree) |  |  |
| High School, equivalent or less |  | 1.806*** (0.096) |
| Some college |  | 1.485*** (0.076) |
| Associate’s degree |  | 1.310*** (0.084) |
| Postgraduate |  | 0.968 (0.067) |
| Employment Status (Ref.: full time) |  |  |
| Self-employed |  | 1.063 (0.071) |
| Work for employer part time |  | 0.934 (0.058) |
| Not working |  | 0.974 (0.042) |
| Annual Income (Ref.: $50,000 to $74,999) |  |  |
| Less than $25,000 |  | 1.300*** (0.075) |
| $25,000 to $49,999 |  | 1.358*** (0.069) |
| $75,000 to $99,999 |  | 0.936 (0.059) |
| $100,000 to $149,999 |  | 0.749*** (0.052) |
| $150,000 or more |  | 0.621*** (0.062) |
| Armed Services (Ref.: Never member) |  |  |
| Current member |  | 4.587*** (0.548) |
| Previous member |  | 1.732*** (0.101) |
| Constant | 0.054*** (0.003) | 0.172*** (0.023) |
| Log likelihood | -12,901.261 | -10,795.556 |
| Pseudo R2 | 0.088 | 0.237 |
|  |  |  |

Notes: N=24,103; ****p*<0.001 ***p*< 0.01 **p*<0.05
